# Integration of HIV prevention into Sexual and Reproductive Health services in an urban setting in South Africa

**DOI:** 10.4102/phcfm.v5i1.522

**Published:** 2013-08-23

**Authors:** Shireen Parker, Vera Scott

**Affiliations:** 1 School of Public Health, University of the Western Cape, South Africa

## Abstract

**Background:**

The United Nations Political Declaration on HIV and AIDS of 2006 stressed the need to strengthen policy and programme linkages between HIV and Sexual and Reproductive Health (SRH). However, the effectiveness and best practices for strengthening SRH and HIV linkages are poorly researched in the context of family-planning services. In Cape Town, HIV-prevention services have been integrated into family-planning services. There are two models of service configuration: dedicated stand-alone reproductive health clinics and family-planning services located in comprehensive primary-care facilities.

**Objective:**

To describe how reproductive health services are integrating HIV prevention and care strategies and to measure the coverage and quality of these integrated services.

**Methods:**

A cross-sectional study was conducted using structured interviews with facility managers; a facility-based checklist; and a patient record review to assess the availability of resources, training, access, quality and integration.

**Results:**

Facilities in Cape Town are equipped adequately to offer integrated HIV-prevention and SRH services. Overall there was poor coverage of integrated services with 54% of family-planning clients having a known HIV status; 47% being screened for a sexually transmitted infection and 55% being offered HIV counselling and testing and receiving condoms. Quality and continuity of care seemed better at the dedicated clinics than at the comprehensive facilities, supported by better training coverage.

**Conclusion:**

Engaging middle-level management is crucial with regard to improving integration within a well-resourced setting.

## Abstrait

**L'intégration de la prévention du VIH aux services de santé sexuelle et reproductive dans un contexte urbain en Afrique du Sud**

**Contexte:**

La Déclaration politique des Nations unies sur le VIH et le Sida de 2006 a mis en avant la nécessité de renforcer les liens des politiques et programmes entre le VIH et la Santé sexuelle et reproductive (SSR). Cependant, l'efficacité et les meilleures pratiques visant à renforcer les liens entre SSR et VIH sont peu étudiées dans le contexte des services de planification familiale. Au Cap, les services de prévention du VIH ont été intégrés aux services de planification familiale. Il existe deux modèles de configuration de service: cliniques de santé reproductive autonomes dédiées et services de planification familiale situés dans des centres de soins primaires polyvalents.

**Objectif:**

Décrire la façon dont les services de santé reproductive s'intègrent aux stratégies de prévention et de prise en charge du VIH et mesurer la couverture et la qualité de ces services intégrés.

**Méthodes:**

Une étude transversale a été réalisée en utilisant des entretiens structurés avec des responsables de centres; une liste de contrôle basée sur le centre; et un examen du dossier des patients afin d’évaluer la disponibilité des ressources, la formation, l'accès, la qualité et l'intégration.

**Résultats:**

Les centres au Cap sont bien équipés pour proposer des services de prévention du VIH et de SSR intégrés. Dans l'ensemble, la couverture des services intégrés était faible, 54% des clients de la planification familiale connaissant leur état sérologique; 47% ayant été dépistés pour une infection sexuellement transmissible et 55% s’étant vus proposer des conseils et un dépistage du VIH et ayant reçu des préservatifs. La qualité et la continuité de la prise en charge semblent meilleures dans les cliniques dédiées que dans les centres polyvalents, soutenues par une meilleure couverture de la formation.

**Conclusion:**

Engager la direction intermédiaire est essentiel eu égard à l'amélioration de l'intégration au sein d'un contexte disposant de ressources suffisantes.

## Introduction

The 1994 International Conference on Population and Development was a milestone in Sexual and Reproductive Health (SRH) programme development. It recognised that achieving universal access to sexual and reproductive health is necessary in order to further national development goals and eradicate Human Immunodeficiency Virus (HIV).^1^ However, in the years that followed, global funding separated HIV programmes from SRH.^2^ A re-commitment to integrated approaches was affirmed in the United Nations Political Declaration on HIV and AIDS of 2006 which saw the need to ‘strengthen policy and programme linkages and coordination between HIV/AIDS, sexual and reproductive health, national development plans and strategies’ in the prevention of HIV infections.^3^

Service integration is the bringing together of different activities that share common goals.^4^ From a programme perspective, a strong argument exists for integrated approaches as both HIV and SRH services have similar target groups, address human sexuality, rely on effective prevention and promotion through the distribution of condoms and use similar medical skills to address sensitive sexual issues. Closer collaboration between the two services will not only be cost effective, but would help to tackle some of the missed opportunities by providing a comprehensive reproductive health service that holistically addresses clients’ dual risks of HIV infection and unintended pregnancy.^5^ In this way, more clients are reached with a broader variety of services.

Integration of services may occur across a range of interventions in a variety of settings with traditional family-planning clinics and antenatal services offering or linking HIV activities or services to a fuller range of reproductive health services.^6^ A systematic literature review was performed by the World Health Organization (WHO) in conjunction with the United Nations Population Fund, Joint United Nations Programme on HIV/AIDS, International Planned Parenthood Federation and the University of California San Francisco in 2009 in order to gain a better understanding of the effectiveness and best practices for strengthening SRH and HIV linkages.^7^ Fifty-eight studies published between 1990 and 2007 met the criteria: 35 studies published in peer-reviewed journals and 23 in grey literature. Of these, only six were conducted in family-planning clinics and none of these were peer reviewed. There is therefore a gap in the peer-reviewed literature that this article hopes to fill. The review found that, in the family-planning setting, grey literature suggests that the integration of HIV services into family-planning services is feasible and does not increase waiting times or decrease quality. Reports in the grey literature published after 2007 claim that extending services at family-planning clinics to include aspects of HIV prevention and care is advantageous as it increases the opportunity for a sexually-active population, especially women, to better understand how to protect themselves and their future children from possible infection by knowing their status,^8^ as well as allowing early diagnosis and referral to treatment and care.^9^ Integration can therefore lead to improved coverage, access to and uptake of key services and improved quality of care. It may even enhance programme effectiveness and efficiency as redundancies in vertical programmes are eliminated and clients’ multiple needs are addressed under one roof.^10^

As is the case in many developing countries, South Africa has historically had a vertical family-planning service, meaning that the services were delivered separately with dedicated management, funding and staff.^11^ It was even delivered separately from other reproductive services. Initial integration objectives in South Africa focused on bringing these components together into one integrated reproductive health package delivered at comprehensive primary care facilities rather than stand-alone reproductive-health clinics.^12^ More recently, in the face of the HIV epidemic in South Africa, integrating HIV and reproductive health services at primary-care level has become a national priority as many of the country's reproductive health clients fall into the priority target group for HIV prevention: they are young, sexually-active women who are at risk of contracting HIV.^13^ The reproductive health service is a well-established and well-utilised service, thus presenting a unique opportunity for HIV prevention and early diagnosis by expanding access to women who perceive themselves as being ‘low risk’.^13, 14, 15, 16^ However, the overall quality of the family-planning service has been reported in some studies as being low.^17, 18^ Also, early attempts at policy development and training to support HIV and reproductive health service integration did not result in the desired implementation. Because monitoring and evaluation of these SRH services is not standardised, little data are available to track implementation effectively.^6^

In Cape Town, programme managers have sought specifically to integrate HIV, TB (tuberculosis) and STI (sexually-transmitted infection) services at facility level since 2003 and have extended this notion of integration across the HIV, TB, STI cluster to include reproductive health services. Guidelines and integrated evaluation tools have been developed for use in HIV, TB and STI services in order to support this integrated approach, promoting access for HIV, TB and STI clients to services across the cluster, which include reproductive health services such as family-planning and PAP (Papanicolau) smears.^19, 20^ Whilst much attention has been focused on offering integrated care to HIV, TB and STI clients, the same is not true for family-planning clients. Data are available to assess the extent to which HIV clients access reproductive health services, but not for assessment of the extent to which reproductive health clients access HIV services.^21^ The purpose of this study is therefore to describe how reproductive health services are integrating HIV prevention in an urban subdistrict in Cape Town, South Africa and to measure the coverage and quality of these integrated services.

### Research method and design

### Setting

The Western subdistrict, with an estimated population of 445 767, is one of eight subdistricts in the Cape metropole. It consists of urban and peri-urban areas with vast socioeconomic disparities between communities, ranging from the wealthiest people living in first-world conditions to the poorest living in conditions similar to the worst found in developing countries.^13^ Data show that about 73% of the population in this subdistrict is dependent on the public sector for their healthcare.^22^

Primary-level healthcare, including reproductive health services, are offered by two administrations: the local and provincial health departments. There are 21 facilities in total, 11 run by the provincial government and 10 by the local authority. There are two models of service configuration: dedicated stand-alone reproductive health clinics administered by the province and family-planning services located in comprehensive primary care facilities, administered by either the province or the local authority. Comprehensive reproductive health care comprises prevention, promotion and management of reproductive health needs (including contraception, STIs and/or HIV and termination of pregnancy [TOP]). Services are delivered primarily by professional nurses, whilst in some clinics, enrolled nurses and nursing assistants provide family-planning services under the supervision of a professional nurse. Mobile and satellite clinics, however, can only be manned by professional nurses or enrolled nurses trained in family-planning.

### Design

This research is based on a descriptive cross-sectional design.

### Procedure

The data-collection tools, consisting of a structured facility manager's questionnaire, consulting room observations and a folder or patient-held record review, were modelled on existing integrated HIV, TB and STI evaluation tools which had been validated in the same setting over a period of five years.^19^ They were piloted at a provincial and a local authority clinic prior to data collection, following which a few minor adjustments were made. Data were collected on the extent to which family-planning clients receive integrated services (defined as PAP smears, STI assessment, HIV counselling and testing [HCT]), as well as the quality of care. The measurement of the quality of reproductive health care encompasses a wider range of variables. For the purposes of this study, quality had been defined to include service delivery according to the current provincial reproductive health protocol and all aspects of service support required to deliver this protocol: appropriately-equipped consulting rooms, functional referral and stock-control mechanisms, availability of key resources, adequate staff training and integration of HIV prevention and care into reproductive health services. A set of data-collection tools was used as described below.

### Inclusion criteria

The study population comprised of women attending public primary-care facilities in the Western subdistrict for reproductive health services. It was not feasible for the researcher to travel to all the facilities in the Western sub-district of the Cape metropole, so six facilities were purposively chosen based on two criteria. Firstly, according to the district's monthly headcounts, they represented the largest providers of reproductive health services in the subdistrict, accounting for more than 75% of the reproductive health clients attending public primary care facilities, each with a monthly headcount greater than 500 clients. Secondly, they represented the different facility types run by the two different administrators: two were dedicated reproductive health facilities (Clinics A and B), two were local authority clinics offering comprehensive primary care (Clinics O and P) and two were community-health centres offering comprehensive primary care (Clinics G and H).

Data were collected between February and October 2011. In each facility the facility manager or her designated assistant filled in a questionnaire designed to collect quantitative information on staffing and training. Relevant service output data reflecting access to services were extracted from the routine health information system. A structured facility and consulting room observation checklist was administered by the researcher to assess for the availability of protocols, guidelines, drugs, equipment and supplies. Finally, a record review was used to extract clinical data on access and quality of care from patient folders and patient-held cards. The Epi Info version 6.04d calculator for a descriptive study (2001, Centers for Disease Control and Prevention & World Health Organization) was used to determine the sample size needed for the record review component of this study. A total of 110 folders and patient-held cards were drawn. The sample was drawn proportionately to the headcount for each of the six facilities, with a minimum of 10 records per facility (this minimum was set so as to feed back some meaningful data to each participating facility). The headcount per facility is shown in Table 1. The resulting sample consisted of 59 folders at dedicated reproductive health clinic A, 11 for dedicated reproductive health clinic B and 10 folders or patient-held cards for each of the remaining four facilities G, H, O and P (see Table 4 further on). The use of patient-held cards and formal folders was inconsistent across the six facilities. Dedicated reproductive health clinic A was the only facility where all the patients had both a folder and a patient-held card. At provincial clinics G and H, folders were only opened for clients who presented for a PAP, TOP, HCT or an STI. Family-planning clients were therefore seen on a patient-held card. During the time preceding the data collection for this study, patient-held cards were substituted with appointment cards because patient-held cards were out of stock. Folders were used at local authority clinics O and P.

**TABLE 1:** Service output indicators drawn from the routine health information system.

Service output indicators	Dedicated Reproductive Health Clinics		Local Authority Clinics		Provincial Clinics	
			
	Clinic A	Clinic B	Clinic O	Clinic P	Clinic G	Clinic H
Number of reproductive health clients in preceding month	2017	2111	603	464	841	1098
HCT performed on a reproductive health client in preceding month	121	102	219	106	183	138
Number of male condoms distributed in a calendar year	19770	32600	294155	229140	350210	398413
Ratio of male to female condoms distributed in a calendar year	21: 1	12: 1	32: 1	210: 1	161: 1	134: 1
Number of IUCDs placed in preceding month	2	11	N/A	N/A	N/A	N/A
Number of PAP smears performed in preceding month	99	35	31	15	29	79
Number of TOP assessments in preceding month	-	-	-	-	-	25

*Source*: Routine health information system (authors’ own construction) -, no statistics recorded; N/A, not applicable

This study used a systematic sampling technique for folder sampling. Starting from a random number, the 10th folder was sampled systematically by the researcher from the previous month's folders until the desired sample size was met. An even spread of folders was thus obtained for the dedicated reproductive health clinics and the local authority clinics.

Patient-held cards were sampled in the provincial clinics. Every third patient was sampled systematically as they exited the consulting room from the time the researcher arrived outside the consulting room until the total required for that facility was obtained.

## Ethical considerations

An application for ethical approval was submitted to the University of the Western Cape Ethics Committee before undertaking this study (study registration number 10/9/12). Permission to conduct the study was obtained from the Provincial Department of Health and the Local Authority. Informed consent was also obtained from the facility managers for their participation in the interview and evaluation of the staff's performance. The researcher obtained verbal consent before examining the patient-held cards by identifying herself and briefly explained the aim of the research. Confidentiality was ensured by storing the collected data in a database which was password protected with access restricted to only the researcher and the supervisor.

## Trustworthiness

The data-collection tools were based on existing integrated HIV and/or TB and/or STI evaluation tools which had been validated in the same setting over a period of five years. It used well-defined domains with questions and indicators constructed specifically to measure programme performance in each domain. The content validity of the tool was strengthened in that it was reviewed by the Metro Districts Acting Reproductive Health Coordinator and piloted at a provincial and a local authority clinic prior to data collection in order to determine the reliability of the tool and to ensure that the study was well planned in concept. Selection bias was reduced through the use of random sampling of the folders and patient-held cards, whilst confounding was addressed through the use of a sufficiently-large sample with a narrow confidence interval. The researcher collected the data personally using a standardised questionnaire thus minimising inconsistencies and information bias. Queries on how to interpret the folder or patients-held records were clarified during the pilot.

The results and management lessons of the facilities in this subdistrict are likely to be generalisable to other large facilities in the other subdistricts of the Cape metropole which have a similar staff component offering the same services, with similar supervision and management structures similar training protocols and a similar client pool of uninsured clients in the public sector.

### Data analyses

Indicators were grouped into domains (availability of resources, capacity, access, quality and integration) which aided the structure of the analysis. A descriptive analysis was performed to determine percentages and confidence intervals for each indicator in each domain. Raw data were entered into Excel 2007 (which was used to calculate the means) and a statistical calculator (used to calculate the confidence intervals). In interpreting the adherence to integrated protocols, the adherence to all or most of the aspects required by protocol was considered ‘good’, whilst ‘satisfactory’ referred to more than 60%. ‘Poor’ refers to less than 60% of the aspects required by protocol to deliver the reproductive health service.

## Results

A total of six clinics was audited, 17 consulting rooms used for reproductive health services were inspected and 90 folders from the dedicated provincial and comprehensive local authority clinics, as well as 20 patient-held cards from the comprehensive provincial facilities, were sampled. All six clinics offered family-planning on a walk-in basis and three of the facilities (clinics B, O and P) also offered consultations on an appointment basis. However, reproductive health clinic B and both local authority clinics (O and P) also provided family-planning on an appointment basis. As shown in Table 1, the provincial dedicated reproductive health clinics A and B had the highest patient loads. Both clinics were located in the central business district, making them convenient for working women. They were also the only facilities which offered a full range of reproductive health services, excluding TOP assessments. These comprised oral and injectable contraception, condom distribution, intrauterine contraception device (IUCD) insertion and PAP smears. None of the others offered IUCD insertion.

All six clinics distributed both male and female condoms, but the dedicated reproductive health clinics (A and B) and one local authority clinic (O) distributed a higher proportion of female condoms than the others. All of the facilities offered PAP smears, but whilst this service was available daily at three facilities (B, H and O) it was only available on pre-determined days of the week at the remaining three facilities. All six facilities claimed to offer TOP as a method of choice for unwanted pregnancies and this service was accessible on a walk-in basis. However, only one of the provincial comprehensive facilities could support this claim with data showing that TOP assessments had been done. One of the local authority clinics and both the provincial clinics offered antiretroviral therapy (ART) and HIV wellness (care before the initiation of ART) every day.

Table 2 illustrates the numbers and proportions of staff trained to deliver the various aspects of reproductive health services. The professional nurses at the dedicated services were all well trained in family-planning, PAPs, TOP assessments and IUCDs. Few professional nurses at the provincial clinics were trained in family-planning, PAPs and TOP assessment and even fewer were trained in IUCDs. Family-planning is also within the scope of enrolled nurses, but few were trained in this.

**TABLE 2:** Staff trained in reproductive health procedures.

Staff trained	Dedicated Reproductive Health Clinics		Local Authority Clinics		Provincial Clinics		Total
				
	Clinic A	Clinic B	Clinic O	Clinic P	Clinic G	Clinic H	
Professional Nurses (including CNPs) working at the facility	3	2	8	4	7	24	**48**
Professional Nurses specifically allocated to the reproductive health clinic (in addition to other responsibilities)	*n* = 3 (100%)	*n* = 2 (100%)	*n* = 8 (100%)	*n* = 4 (100%)	*n* = 1 (14%)	*n* = 1 (4%)	**19 (40%)**
Professional Nurses trained in family-planning	*n* = 3 (100%)	*n* = 2 (100%)	*n* = 1 (13%)	*n* = 1 (25%)	*n* = 0 (0%)	*n* = 6 (25%)	**13 (27%)**
Professional Nurses trained to insert an IUCD	*n* = 0 (0%)	*n* = 2 (100%)	*n* = 1 (13%)	*n* = 0 (0%)	*n* = 0 (0%)	*n* = 1 (4%)	**4 (8%)**
Professional Nurses trained in TOP assessment	*n* = 3 (100%)	*n* = 2 (100%)	*n* = 8 (100%)	*n* = 0 (0%)	*n* = 1 (14%)	*n* = 4 (17%)	**18 (38%)**
Professional Nurses trained in PAPs	*n* = 3 (100%)	*n* = 2 (100%)	*n* = 6 (75%)	*n* = 4 (100%)	*n* = 1 (14%)	*n* = 3 (13%)	**19 (40%)**
Enrolled Nurses working at the facility	1	2	1	1	2	23	**30**
Enrolled Nurses trained in family-planning	*n* = 0 (0%)	*n* = 2 (100%)	*n* = 0 (0%)	*n* = 1 (100%)	*n* = 0 (0%)	*n* = 1 (4%)	**4 (13%)**

*Source*: Structured interview with facility managers CNP, Clinical nurse practitioner; IUCD, intra-uterine contraceptive device; TOP, termination of pregnancy; PAP, Papanicolou smear.

**FIGURE 1:**
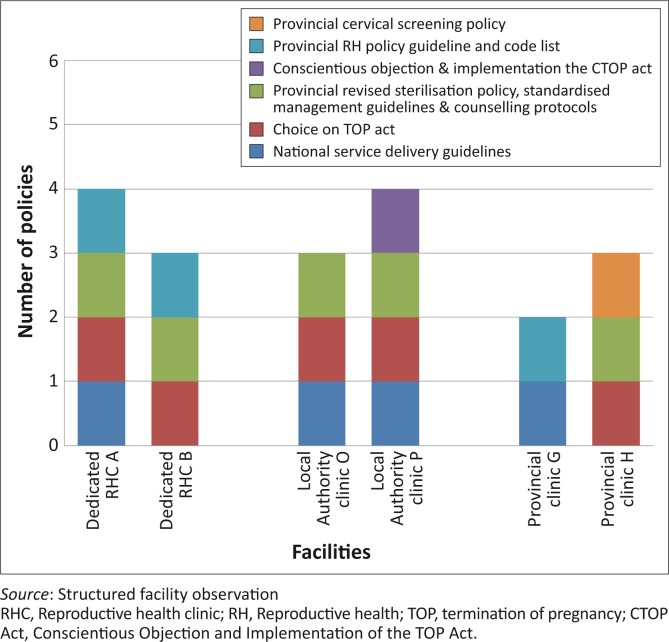
Policy documents, guidelines & Acts accessible in the facility manager's office (maximum = 6).

As shown in Figure 1, none of the facilities had all the stipulated policies, guidelines or Acts available in the facility manager's office. The Provincial Cervical Screening Policy and the Conscientious Objection and Implementation of the TOP Act were the two policies least available. Managers reported that even though new policies were discussed in meetings, very few documents were forwarded to facility level. Facility managers often had to take it upon themselves to access documents in order to store them on file for reference in the facility.

Table 3 shows that only local authority clinic P had integrated reproductive health services exclusively into the general consultation setting; the other five still made use of dedicated reproductive health consulting rooms. However, even clinic P still did not have all clinical consulting rooms set up to offer family-planning, PAP and TOP assessments.

**TABLE 3:** Structured consulting room observations (*n* = 17).

Variables	Dedicated Reproductive Health Clinics		Local Authority Clinics		Provinci al Clinics		Total
				
	Clinic A	Clinic B	Clinic O	Clinic P	Clinic G	Clinic H	
**Structured consulting room observations**							
Number of clinical consulting rooms at the clinic	6	2	12	4	7	13	**44**
Presence of a dedicated family-planning room	Yes	Yes	Yes	No	Yes	Yes	
Clinical consulting rooms used for family-planning (only or together with other services)	*n* = 4 (67%)	*n* = 2 (100%)	*n* = 6 (50%)	*n* = 3 (75%)	*n* = 1 (14%)	*n* = 1 (8%)	**17 (39%)**
Clinical consulting rooms used for PAP smears (only or together with other services)	*n* = 4 (67%)	*n* = 2 (100%)	*n* = 5 (42%)	*n* = 3 (75%)	*n* = 1 (14%)	*n* = 5 (38%)	**20 (45%)**
Clinical consulting rooms used for TOP assessment (only or together with other services)	*n* = 5 (83%)	*n* = 2 (100%)	*n* = 7 (58%)	*n* = 3 (75%)	*n* = 1 (14%)	*n* = 1 (8%)	**19 (43%)**
**Documentation, Quick References & Educational leaflets available in the consulting or counselling room**							
Clinical consulting rooms used for reproductive health	4	2	6	3	1	1	***17***
Quick STI treatment references available	*n* = 4 (100%)	*n* = 2 (100%)	*n* = 5 (83%)	*n* = 3 (100%)	*n* = 1 (100%)	*n* = 1 (100%)	**16 (94%)**
Reproductive health educational leaflets available	*n* = 4 (100%)	*n* = 2 (100%)	*n* = 2 (33%)	*n* = 3 (100%)	*n* = 1 (100%)	*n* = 1 (100%)	**13 (76%)**
Sterilisation contact information for clients available	*n* = 4 (100%)	*n* = 2 (100%)	*n* = 4 (67%)	*n* = 3 (100%)	*n* = 1 (100%)	*n* = 1 (100%)	**15 (88%)**
Female sterilisation assessment forms available	*n* = 4 (100%)	*n* = 2 (100%)	*n* = 4 (67%)	*n* = 3 (100%)	*n* = 1 (100%)	*n* = 1 (100%)	**15 (88%)**
Other forms – TOP assessment, specific stationery or consent forms available	*n* = 4 (100%)	*n* = 2 (100%)	*n* = 4 (67%)	*n* = 3 (100%)	*n* = 1 (100%)	*n* = 1 (100%)	**15 (88%)**
**Privacy & Equipment available in the consulting room**							
Privacy (Restricted access/lockable door/screen)	*n* = 4 (100%)	*n* = 2 (100%)	*n* = 6 (100%)	*n* = 3 (100%)	*n* = 1 (100%)	*n* = 1 (100%)	**17 (100%)**
Examination couch	*n* = 4 (100%)	*n* = 2 (100%)	*n* = 6 (100%)	*n* = 3 (100%)	*n* = 1 (100%)	*n* = 1 (100%)	**17 (100%)**
Fixed or mobile angle-poised lamp	*n* = 4 (100%)	*n* = 2 (100%)	*n* = 4 (67%)	*n* = 3 (100%)	*n* = 1 (100%)	*n* = 1 (100%)	**15 (88%)**
Three different size specula	*n* = 4 (100%)	*n* = 2 (100%)	*n* = 2 (33%)	*n* = 3 (100%)	*n* = 1 (100%)	*n* = 1 (100%)	**13 (76%)**
Cervical brushes, slides and fixatives	*n* = 4 (100%)	*n* = 2 (100%)	*n* = 2 (33%)	*n* = 3 (100%)	*n* = 1 (100%)	*n* = 1 (100%)	**13 (76%)**
Dildo	*n* = 4 (100%)	*n* = 2 (100%)	*n* = 1 (17%)	*n* = 3 (100%)	*n* = 1 (100%)	*n* = 1 (100%)	**12 (71%)**
Male condoms	*n* = 4 (100%)	*n* = 2 (100%)	*n* = 6 (100%)	*n* = 3 (100%)	*n* = 1 (100%)	*n* = 1 (100%)	**17 (100%)**
Female condoms	*n* = 2 (50%)	*n* = 2 (100%)	*n* = 6 (100%)	*n* = 0 (0%)	*n* = 1 (100%)	*n* = 1 (100%)	**12 (71%)**

*Source*: Structured consulting room observations PAP, Papanicolou smear; TOP, termination of pregnancy; STI, sexually-transmitted infection.

Table 3 also shows that all the consulting rooms used for reproductive health in each facility (except for local authority clinic O) were sufficiently private, had examination couches and were equipped adequately with lamps, speculae, cervical brushes and fixatives, dildos and male condoms in order to perform reproductive health services. Female condoms were not available in all rooms in Clinic A and were available in none of the rooms in Clinic P. Every facility (although not necessarily each consulting room used for reproductive health services in the facility) had a functioning weighing scale, blood-pressure machine and a PAP register. Only the two facilities which offered IUCDs had insertion kits available. Table 4 shows that in each facility, except Clinic O, all of the consulting rooms used for reproductive health had the required stationery: quick STI treatment references, reproductive health educational leaflets, sterilisation contact information and assessment forms, TOP assessment forms and consent forms.

**TABLE 4:** Client record review (*n* = 110).

Variables	Dedicated Reproductive Health Clinics		Local Authority Clinics		Provincial Clinics		Total
				
	Clinic A (*n* = 59)	Clinic B (*n* = 11)	Clinic O (*n* = 10)	Clinic P (*n* = 10)	Clinic G (*n* = 10)	Clinic H (*n* = 10)	110
**Quality and continuity of services rendered**							
Date of birth recorded	100% (CI 94–99)	100% (CI 74–99)	100% (ci 72–99)	100% (CI 72–99)	0% (CI 0–28)	70% (CI 39–89)	**88% (CI 80–93)**
Parity of client recorded	95% (CI 86–98)	100% (CI 74–99)	80% (CI 48–94)	100% (CI 72–99)	0% (CI 0–28)	10% (CI 2–41)	**78% (CI 70–85)**
Client's weight recorded	*n* = 58 (98%) (CI 91–99)	*n* = 10 (91%) (CI 62–98)	*n* = 9 (90%) (CI 59–98)	*n* = 9 (90%) (CI 59–98)	*n* = 4 (40%) (CI 17–69)	*n* = 1 (10%) (CI 2–41)	**91 (83%) (CI 75–88)**
Client's blood pressure recorded	*n* = 59 (100%) (CI 94–99)	*n* = 10 (91%) (CI 62–98)	*n* = 7 (70%) (CI 39–89)	*n* = 4 (40%) (CI 17–69)	*n* = 5 (50%) (CI 23–77)	*n* = 1 (10%) (CI 2–41)	**86 (78%) (CI 70–85)**
Safer sex discussion recorded	*n* = 51 (86%) (CI 75–93)	*n* = 9 (82%) (CI 52–95)	*n* = 4 (40%) (CI 17–69)	*n* = 4 (40%) (CI 17–69)	*n* = 0 (0%) (CI 0–28)	*n* = 0 (0%) (CI 0–28)	**68 (62%) (CI 52–70)**
Date of the last PAP smear recorded*	*n* = 27/31 (87%) (CI 71–95)	*n* = 11/11 (100%) (CI 74–99)	*n* = 0/3 (0%) (CI 0–60)	*n* = 2/6 (33%) (CI 6–52)	*n* = 0/10 (0%) (CI 0–28)	*n* = 0/10 (0%) (CI 0–28)	**40/71 (56%) (CI 45–67)**
Return appointment date recorded	*n* = 58 (98%) (CI 91–99)	*n* = 11 (100%) (CI 74–99)	*n* = 10 (100%) (CI 72–99)	*n* = 10 (100%) (CI 72–99)	*n* = 10 (100%) (CI 72–99)	*n* = 10 (100%) (CI 72–99)	**109 (99%) (CI 95–99)**
**Integration of reproductive health and HIV prevention and care**							
Confirmed HIV status (positive or negative)	*n* = 46 (78%) (CI 66–86)	*n* = 8 (73%) (CI 43–90)	*n* = 3 (30%) (CI 11–61)	*n* = 1 (10%) (CI 2–41)	*n* = 0 (0%) (CI 0–28)	*n* = 1 (10%) (CI 2–41)	**59 (54%) (CI 44–63)**
HCT offered**	*n* = 46/57 (81%) (CI 69–89)	*n* = 6/9 (67%) (CI 35–88)	*n* = 4/10 (40%) (CI 17–69)	*n* = 1/9 (11%) (CI 3–45)	*n* = 0/10 (0%) (CI 0–28)	*n* = 1/10 (10%) (CI 2–41)	**58/105 (55%) (CI 43–62)**
Condoms issued	*n* = 46 (78%) (CI 66–86)	*n* = 7 (64%) (CI 34–85)	*n* = 6 (60%) (CI 31–83)	*n* = 1 (10%) (CI 2–41)	*n* = 1 (10%) (CI 2–41)	*n* = 0 (0%) (CI 0–28)	**61 (55%) (CI 46–64)**
Symptomatic screening for STI	*n* = 38 (64%) (CI 52–75)	*n* = 7 (64%) (CI 34–85)	*n* = 4 (40%) (CI 17–69)	*n* = 3 (30%) (CI 11–61)	*n* = 0 (0%) (CI 0–28)	*n* = 0 (0%) (CI 0–28)	**52 (47%) (CI 38–57)**
HIV negative or status unknown client whose had a PAP done in accordance with provincial protocol**	*n* = 52/57 (91%) (CI 81–96)	*n* = 8/9 (89%) (CI 55–97)	*n* = 7/10 (70%) (CI 39–89)	*n* = 5/9 (56%) (CI 26–81)	*n* = [Missing data]/10 [?%] (CI -)	*n* = [Missing data]/10 [?%] (CI -)	**72/105 (69%) (CI 59–77)**

*Source*: Client record reviews PAP, Papanicolou smear; HCT, HIV counselling and treatment; STI, sexually-transmitted infection; - Confidence interval (CI) could not be calculated; N/A not applicable.

* Not every patient qualified for a PAP, either because of age or HIV status.

** HCT offered and the number of HIV negative or status-unknown clients who had a PAP performed in accordance with provincial protocol are subsets of the total.

The record review showed marked differences between facilities in the documentation of services rendered. Generally, services were documented better in folders than in the patient-held cards. Continuity of care requires good documentation and, within the research site, senior managers have stated that a lack of documentation is taken to mean that the service was not rendered and equated it to a poor-quality service. The smaller sample sizes at facilities other than clinic A produced large confidence intervals which need to be interpreted in conjunction with the results. Date of birth, parity and weight were fairly well recorded (88%, 78% and 83% respectively), with higher attainment at the facilities with folders, but not at the facilities with only patient-held cards. Blood pressure, discussion of safer sex and date of last PAP smear were only recorded well at clinics A and B. A return appointment date was recorded at all facilities.

Table 4 illustrates that there was good integration overall of HIV prevention and care measures with reproductive health services at the dedicated clinics and generally poor integration at the other four facilities, except for local authority clinic O, where there was a high distribution of male condoms and PAPs were performed in accordance with provincial policy. Very little data were recorded on the appointment cards which were used as a substitute for the patient-held cards at the provincial clinics. This made assessment of integration very difficult. Just over half of the clients had a confirmed HIV status noted, were offered HCT and were issued with condoms. Symptomatic screening for STIs was performed and recorded in 47% of clients and 69% had a PAP smear performed according to protocol. Generally, the dedicated clinics A and B performed better than the comprehensive facilities.

## Discussion

In this study, two different models of SRH service delivery were operational: stand-alone reproductive health clinics and comprehensive primary care facilities offering SRH. The former is perhaps a historical vestige of the vertical family-planning services, but has been retained because of efficiency considerations – as this study shows, they have high reproductive health service output indicators. HCT was integrated into both of these models. Interestingly the comprehensive primary care facilities still favoured the use of dedicated SRH rooms over integrating SRH services into primary care as offered in all the consulting rooms. This meant that a form of internal referral was required within the facility in order to access the range of SRH activities available. Staff training at the dedicated clinics was more comprehensive, although one dedicated facility had no-one trained in IUCD insertion. In the comprehensive facilities, training was incomplete and irregular, perhaps because the use of dedicated rooms means that universal training is not required. However, in one provincial facility there was no-one trained in family-planning methods and in one local authority facility no-one was trained in TOP assessment. This would have the effect of limiting access to and quality of service. In general, the range of SRH activities readily available in all facilities included oral and injectable contraception, male condoms, PAP smears and HCT. IUCD insertion, TOP assessment and female condoms were only available at certain facilities.

Tracers of quality care assessed in this study included weight recorded (83%), blood-pressure recorded (78%) and safer sex discussed (62%). Church and Mayhew^23^ cautioned that integration has the potential to ‘improve quality of care by increasing the breadth of care provided, or to diminish quality as breadth is achieved at the expense of depth’. To investigate the effect of integration on quality of care, a before–after study design is required. This cross-sectional study presents only a snap shot after integration, but it is interesting to note that the quality of care in this study was better than that noted by Matizirofa.^17^ The sample size for the record review was powered to allow a description of the quality and integration of care received by clients across the six largest facilities in the subdistrict and was not large enough to allow for comparison between the different types of facilities and to support statistical tests. However, some trends are noteworthy and should be the subject of further enquiry. The coverage of HIV prevention activities and the quality of care at the dedicated clinics seems to be better than at the comprehensive facilities. This might be related to the higher coverage of training on all aspects of SRH, or to the facilities’ dedicated focus on SRH, whilst managers in the comprehensive facilities are responsible for a wider range of programmes which all demand management time and attention. Both dedicated and comprehensive facilities had rooms equipped to support a quality consultation, although there were instances where the female condom was not available – a missed opportunity in promoting dual protection. A study by Chersich and Rees^24^ suggests that, were female condoms widely available and at a low cost, many more women would be able to protect themselves from HIV and other STIs.

Overall there was poor integration of services, with only about half of the clients having a known HIV status, being offered HCT, receiving condoms and being screened for an STI. Again, the level of integration at the dedicated clinics appears to be better than at the comprehensive facilities, although a larger sample size would be required to confirm this. The poor level of integration raises an interesting question: why, within a service which intends to be integrated and which actively promotes integration of HIV, TB, STI and SRH, are higher levels of integration not achieved? Policy guidelines are in place and the intention is that clients are offered HCT every six months. The HCT consent form prompts the service provider to screen symptomatically for STIs and to perform a PAP smear according to protocol. In this setting, there may be an unintended tension between the twin goals of increasing access to SRH and increasing quality and integration of services. Long waits in queues and times spent in facilities are acknowledged commonly as being a deterrent to healthy clients receiving a preventative service. In this study setting, various strategies have been implemented so as to reduce the client waiting times: the so-called ‘fast-track’, which streamlines the process from reception to consulting room and the continued use of dedicated consulting rooms. Within this context, staff may feel pressurised to keep the consultation time short so that clients don't have to wait too long. This, however, clashes with the policy which offers a quality, integrated SRH service, giving clients access to a sweep of services within the facility.

Another factor which is likely to reduce integrated care in this study setting is that none of the facilities offered integration at the level of the provider; instead integration was intended to happen at the facility level. Integrated care at the facility level, implying an internal referral, may or may not occur at the time of the visit. Maharaj and Cleland^11^ reported an understandable and perhaps inevitable link that existed between the size of the facility (number of services) and the way in which integrated services are offered, especially where there are referrals within a facility: the smaller the staff complement, the more likely the facility was to offer a purer or more extreme form of responsive integration, with patients in a single queue or waiting line and no specialisation between staff members, whereas a degree of specialisation remained amongst staff members at the bigger facilities, with separate queues or clinics for family-planning, HIV prevention and care and basic curative care. Referral models where reproductive health and HIV prevention and care are not integrated have to maintain effective linkages between services in order to ensure sufficient coverage and continuity of care.^23^ Research has shown that referral services, be they on-site or at another facility, are usually prone to potential loss to follow-up as clients are reluctant to wait for a second consultation.^25^ This internal referral mechanism could explain why there are so many missed opportunities, particularly at the local authority and provincial clinics.

A literature review by Church and Mayhew^23^ which included 44 studies (mainly in sub-Saharan Africa), found several studies which identified poor or insufficient training as being an important constraint to the provision of integrated SRH and HIV services. Another review by Rowe et al.^26^ focused more broadly on how to improve performance of health workers in low-resource settings. They found that training together with supervision was effective, as was supervision and audit with feedback, whilst dissemination of guidelines without additional interventions was generally ineffective and supervision on its own only improved performance on a short-term basis. Within the South African context, Doherty et al.^27^ and Scott et al.^20^ have demonstrated the use of a participatory approach to routine audits as part of a quality improvement strategy: middle-level managers have been engaged to conduct their own programme assessment and to interpret and act on the findings. This process is performed on a periodic and continual basis. Corrective measures taken are followed up with another audit in order to determine if the problem has been solved. In the TB programme in Cape Town, this approach has been found to bring about significant improvements in integrating HIV and TB by equipping middle management with information for local health system strengthening and providing supportive supervision whilst working toward measurable outputs.^28^ The assessment tools developed and used in this study are suitable for use in such a quality improvement process.

## Limitations

A key limitation of this study was that the assessment of quality and integration of care relied on record review, thus the format, completeness and accuracy of the information could have been compromised. It may well be that the quality and extent of integration is greater than reported. However, good record keeping is in itself an important aspect of quality with regard to the improvement of continuity of care.

Another limitation to this study was that it did not look at the ongoing training, mentoring and supervision, but rather just at whether staff had received any of the recognised training. The assessment tool is limited to the service outputs of a quality service which adheres to protocols. It does not include an assessment of what the service recipients, the clients, think of the service, which is an important aspect of quality. If clients’ expectations are to be met, it is important for communities to be able to voice their opinion on the way in which health services are designed and operate.

Finally, the subset of HIV-positive clients included in the sample and the number of fully-comprehensive primary healthcare facilities compared with the dedicated reproductive health clinics was too small to allow for meaningful interpretation.

## Conclusion

Integration of HIV prevention activities into family-planning services at a facility level is feasible within the current setting. Factors favouring integration include the fact that reproductive health, family-planning and HIV prevention and care services are offered within the same facility on a daily basis; adequately-trained staff; appropriately-equipped consulting rooms; adequate infrastructure; an availability of key resources; and a functional referral and stock-control mechanism in place. Overall, there was poor integration of services even though the integration of HIV and reproductive health services at primary-care level is a national priority. The coverage of HIV-prevention activities and the quality of care at the dedicated clinics seemed to be better than at the comprehensive facilities. This is in all likelihood a result of better SRH training coverage, less internal referral between service points and more focused management with regard to SRH. In this setting, there may be an unintended tension between the twin goals of increasing access to HIV prevention and care and increasing the quality and integration of these services. Engaging middle management is therefore crucial if a balance is to be achieved between quality of care and access via integrated care.
